# The Liver-Brain Axis of Alcohol-Mediated Neurodegeneration: Role of Toxic Lipids

**DOI:** 10.3390/ijerph6072055

**Published:** 2009-07-23

**Authors:** Suzanne M. de la Monte, Lisa Longato, Ming Tong, Sarah DeNucci, Jack R. Wands

**Affiliations:** 1 Division of Neuropathology, Brown University, Providence, RI, USA; 2 Division of Gastroenterology, Brown University, Providence, RI, USA; 3 Department of Medicine, Brown University, Providence, RI, USA; 4 Department of Pathology, Brown University, Providence, RI, USA; 5 Department of Neurology, Brown University, Providence, RI, USA; 6 The Liver Research Center, Brown University, Providence, RI, USA; 7 Rhode Island Hospital, Brown University, Providence, RI, USA; 8 The Warren Alpert Medical School, Brown University, Providence, RI, USA

**Keywords:** ethanol, insulin resistance, ceramides, neurodegeneration, alcoholic liver disease, steatohepatitis

## Abstract

Alcohol abuse causes progressive toxicity and degeneration in liver and brain due to insulin resistance, which exacerbates oxidative stress and pro-inflammatory cytokine activation. Alcohol-induced steatohepatitis promotes synthesis and accumulation of ceramides and other toxic lipids that cause insulin resistance. Ceramides can readily cross the blood-brain barrier, and ceramide exposure causes neurodegeneration with insulin resistance and oxidative stress, similar to the effects of alcohol. Therefore, in addition to its direct neurotoxic effects, alcohol misuse establishes a liver-brain axis of neurodegeneration mediated by toxic lipid trafficking across the blood-brain barrier, leading to progressive white matter degeneration and cognitive impairment.

## 1. Introduction-Alcoholic Degeneration in Liver and Brain

### Alcoholic Liver and Brain Diseases

Alcohol dependence and abuse are among the most costly healthcare problems in the world, and their impact continues to grow due to the rising incidence of heavy alcohol drinking among women and young people. Excessive drinking can cause chronic liver injury associated with profound impairments in hepatocellular regeneration [1–4]. Cirrhosis develops when recurrent injury and cell loss are not adequately counterbalanced by repair mechanisms, due to inhibition of DNA synthesis [5–10], reduced energy metabolism, insulin resistance, and oxidative stress [11,12].

The central nervous system (CNS) is the other major target of alcohol toxicity and degeneration. Chronic alcohol abuse causes cognitive impairment with permanent structural damage to the brain. Although Wernicke-Korsakoff syndrome is one of the most devastating forms of alcohol-associated neurodegeneration, its pathogenesis is largely related to thiamine deficiency [13,14]. In contrast, more common alcohol-related brain lesions, including white matter attrition/degeneration (leukoencephalopathy), ventriculomegaly, cerebellar degeneration, and neuronal loss in the superior frontal association cortex, anterior cingulate region, hippocampus, entorhinal cortex, and hypothalamus, which contribute to cognitive and motor deficits [13–15], are still under investigation, although evidence suggests that these pathologies are mediated by insulin resistance [16].

## 2. Effects of Alcohol on Insulin and Insulin-like Growth Factor Signaling

### Insulin and IGF-1 Actions in Liver and Brain:

Insulin transmits pro-growth and pro-survival signals by activating complex intracellular pathways, beginning with ligand binding to cell surface receptors. Subsequent activation of intrinsic receptor tyrosine kinases results in tyrosine phosphorylation of the insulin receptor substrate, type 1 (IRS-1), which transmits signals downstream to promote growth, survival, and energy metabolism [17]. A critical feature of the insulin/IRS signal transduction cascade is the interaction of tyrosyl phosphorylated (PY) IRS-1 with adaptor molecules that contain *src* homology domains, such as Grb2 and the p85 subunit of PI3 kinase, to promote mitogenesis, cell survival, gene expression, energy metabolism, and motility, all of which are needed for liver remodeling and repair after injury [17–19]. A very similar signaling pathway exists for insulin-like growth factor type 1 (IGF-1), but at physiological concentrations, insulin and IGF-1 selectively bind to their own receptors to mediate distinct functions.

In the CNS, insulin/IGF-1 signaling cascades have critical roles in regulating and maintaining cognitive and motor functions. Insulin, IGF-1 and IGF-2, and their corresponding receptors are abundantly expressed in various cell types throughout the brain, including neurons [17,20,21] and oligodendroglia [22–24]. Insulin and IGF signaling pathways utilized by CNS neurons are virtually identical to those present in liver, except that IRS-2 instead of IRS-1 is the major docking protein [17,25]. The highest levels of insulin and IGF polypeptide and receptor gene expression are distributed in the hypothalamus, temporal lobe, and cerebellum [17], i.e., major targets of ethanol-mediated neurotoxicity. Since insulin and IGF mediate neuronal and/or oligodendroglial survival, plasticity, energy metabolism, and neurotransmitter function [17,26–29], sustained impairments in their corresponding signal transduction cascades would have dire consequences with respect to cognition and behavior.

### Ethanol-Mediated Liver Injury and Degeneration Linked to Inhibition of Insulin and IGF Signaling

Alcohol-induced liver injury is associated with steatohepatitis, which is largely reversible when alcohol abuse is stopped. Otherwise, steatohepatitis may progress through stages of fibrosis, followed by cirrhosis and then end-stage liver disease. Superimposed insults, such as obesity, metabolic syndrome, infections with Hepatitis B (HBV) or Hepatitis C (HCV) virus, or hemochromatosis, can exacerbate alcoholic liver disease and cause it to progress. Alcohol mediates its adverse effects by inhibiting DNA synthesis and thereby impairing the liver’s capacity to regenerate [3,4,30]. In addition, repair and survival mechanisms become compromised by increased oxidative stress, DNA damage, mitochondrial dysfunction, lipid peroxidation, and pro-inflammatory cytokine activation [12,31,32]. These anti-growth and pro-stress cascades are driven, in part, by ethanol’s inhibitory effects on insulin and IGF-1 signaling [8–11,31]. As the populations of insulin or IGF-1 receptor bearing hepatocytes decline, insulin/IGF-1 responsive gene expression and functions deteriorate [11,12].

We have characterized the effects of chronic ethanol feeding in three rat strains that we found to be distinguished by their inherently low (Fisher 344; FS), intermediate (Sprague-Dawley; SD), or high (Long Evans; LE) levels of susceptibility to alcohol-induced liver injury. After eight weeks of chronic ethanol feeding (37% caloric content, liquid diet), FS rats had minimal injury and hepatic steatosis, SD rats had patchy micro- and macro-steatohepatitis with early fibrosis, and LE rats had conspicuous micro- and macro-steatohepatitis with apoptotic bodies, disorganized hepatic chord architecture, and chickenwire (peri-hepatocyte) fibrosis [12].

**Figure 1.**
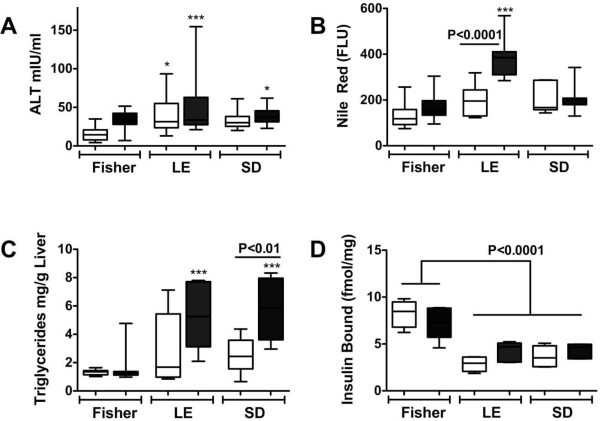
Genetic factors govern susceptibility to alcohol-induced liver injury, steatosis, and insulin resistance. Fisher 344 (Fisher), Long Evans (LE), and Sprague Dawley (SD) rats were fed with control or ethanol containing liquid diets for eight weeks, and then (A) serum was obtained to measure to measure alanine aminotransferase (ALT), and livers were harvested to assess (B) lipid content using the Nile Red assay, (C) triglycerides, and (D) insulin receptor binding. (* P < 0.05; *** P < 0.001; FLU = fluorescent light units) [11,12].

Biochemical and protein assays showed that ethanol-fed LE rats had the highest hepatic levels of neutral lipids and triglycerides, and the lowest levels of insulin receptor binding, while SD rats had intermediate values, and FS had the lowest hepatic lipid content and significantly higher levels of insulin receptor binding compared with the other two strains (Figure 1). In addition, we noted that control LE rat livers had reduced insulin receptor binding relative to control SD and FS rats [12], indicating that genetic factors most likely establish thresholds for hepatic insulin resistance, a phenomenon that could account for differences in host susceptibility to alcohol-induced liver injury.

## 3. Ethanol-Mediated Insulin Resistance-Mechanisms and Consequences

### Chronic Ethanol Abuse Causes Brain Insulin Resistance

Chronic ethanol exposure causes apoptotic and mitochondrial death of insulin/IGF expressing and insulin/IGF receptor bearing cells in the brain [16,33,34]. In addition, ethanol causes insulin/IGF resistance by impairing receptor binding [16,35,36]. These adverse effects of ethanol are dose-dependent. In adult rats and humans, ethanol-induced neurodegeneration in the cerebellum, anterior cingulate gyrus (frontal lobe), hypothalamus, and temporal lobe is associated with the same molecular and biochemical abnormalities demonstrated in liver, including insulin and IGF-1 resistance and mitochondrial dysfunction [16,35]. Importantly, alcohol-mediated neurodegeneration results in loss of neurons and oligodendroglia, decreased mRNA levels of myelin-associated, insulin-responsive, and mitochondrial genes, and increased indices of apoptosis, oxidative stress, mitochondrial dysfunction, lipid peroxidation, DNA damage, and deregulated acetylcholine homeostasis [35].

**Figure 2.**
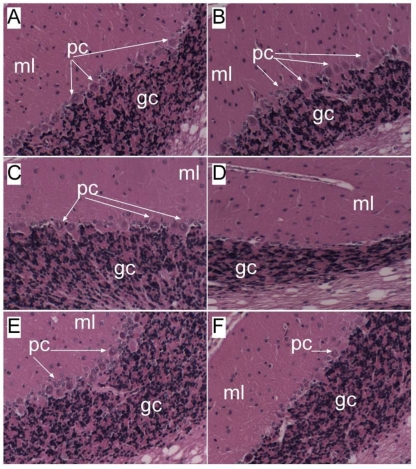
Genetic factors regulate severity of alcohol-induced neurodegeneration. Cerebella of Fisher (A,B), LE (C,D), and SD (E,F) control (A,C,E) or ethanol (B,D,F) fed rats were examined histologically. Control cerebella in each group had a normal tri-laminar architecture with intact molecular layer (ml), Purkinje layer (pc and arrows), and granule cell (gc) layers. Ethanol feeding had minimal effects on Fisher rat cerebella (B), but caused severe degeneration of Purkinje cell and granule cell layers in LE greater than SD rats [16,35,37].

Analysis of temporal lobes and cerebella from LE, SD, and FS rats revealed similar trends with respect to severity of alcohol-induced neurodegeneration (Figure 2), including brain insulin resistance, oxidative stress, and neuronal and oligodendroglial cell loss. However, unlike liver, the control rats did not differ by strain with respect to baseline levels of insulin and IGF receptor binding in brain. Therefore, increased susceptibility to alcohol-mediated neurodegeneration correlates with inherent strain differences in the levels of insulin/IGF resistance in liver, and the degrees to which ethanol increases insulin/IGF resistance in liver and brain-- supporting the concept of a liver-brain axis of neurodegeneration.

### Signaling Pathways Mediating Hepatic and Brain Insulin Resistance

Research conducted over the past 15 years has demonstrated that ethanol mediates its adverse effects on hepatocytes and liver and brain through inhibition of insulin and IGF signaling mechanisms [37]. In vitro and in vivo experiments revealed that ethanol inhibits hepatocellular insulin signaling by reducing insulin receptor binding, insulin receptor tyrosine phosphorylation, and activation of intrinsic receptor tyrosine kinase. In essence, chronic ethanol exposure causes hepatic insulin/IGF-1 resistance [38–43]. Correspondingly, Erk MAPK activation of DNA synthesis and liver regeneration [5,8–10], and phosphatidylinositol-3-kinase (PI3 Kinase) stimulation of cell growth, survival, glucose utilization, and energy metabolism are impaired by chronic ethanol feeding [8,31,44–48]. Therefore, chronic ethanol exposure constitutively increases hepatocellular injury, DNA damage, and pro-apoptosis mechanisms [31,49,50], while inhibiting insulin/IGF-1 stimulated pathways responsible for survival, energy metabolism, regeneration, remodeling, and repair.

Chronic ethanol exposure also inhibits insulin and IGF signaling mechanisms in the CNS [51–54]. As described for liver, ethanol mediates its adverse effects in the CNS by impairing binding to cell surface receptors, reducing receptor tyrosine kinase activation, and inhibiting downstream signaling through IRS, including activation of insulin/IGF responsive genes [34,36]. In addition, ethanol causes oxidative stress, DNA damage, lipid peroxidation, and mitochondrial dysfunction, in part due to inhibition of insulin signaling, but also via acetaldehyde-mediated adduct formation [55]. In contrast to liver, in the CNS, ethanol disproportionately impairs signaling through PI3 kinase-Akt [33,52,54,56]. Correspondingly, major adverse effects of ethanol on CNS neurons include, reduced survival and plasticity, increased apoptosis [52,54,57], and mitochondrial dysfunction with deficits in energy metabolism and acetylcholine homeostasis [33,52,56,58]. The lopsided inhibition of PI3K-Akt and attendant activation of glycogen synthase kinase 3β (GSK-3β) in neurons and brain is partly due to ethanol-activation of phosphatases such as PTP-1b and PTEN [59].

### Ethanol-Induced Brain Insulin/IGF Resistance Linked to Altered Membrane Lipid Composition

Insulin and IGF signaling are mediated by ligand binding to membrane-anchored receptors. Since significant alterations in membrane lipid composition can impair receptor binding and subsequent downstream signaling [60,61], we tested the hypothesis that ethanol-induced insulin/IGF resistance in brain is mediated by altered membrane lipid composition [36]. Our experimental results showed that: 1) neuronal membrane cholesterol content is reduced by ethanol exposure; 2) methyl-β-cyclodextrin (MβCD) depletion of membrane cholesterol impairs insulin receptor binding and insulin-stimulated glucose uptake; and 3) cholesterol repletion partially restores insulin receptor binding and glucose uptake in ethanol-exposed neuronal cells. Therefore, ethanol-induced perturbations in membrane lipid composition contribute to insulin/IGF resistance in brain. However, cholesterol repletion did not fully restore insulin/IGF-1 responsiveness in ethanol-exposed neurons. One possible explanation is that, besides cholesterol, ethanol depletes a variety of membrane lipids, including sphingomyelin. Correspondingly, recent studies also demonstrated that ethanol-induced hepatic insulin resistance was associated with altered expression of genes utilized in lipid metabolism, including both synthetic and degradative pathways.

## 4. Ceramides Mediate Insulin Resistance

### Steatohepatitis, Insulin Resistance, and Toxic Lipid Generation

To determine if hepatic insulin resistance was a consequence of hepatic steatosis, we measured insulin and IGF receptor binding, gene expression, and signaling in livers from several experimental models in which hepatic steatosis or steatohepatitis is a prominent feature including: 1) chronic ethanol feeding (alcohol-induced steatohepatitis; ASH); 2) diet induced obesity (DIO) with non-alcoholic steatohepatitis (NASH); 3) high fat diet (HFD) feeding without obesity; 4) nitrosamine-mediated injury; and 5) constitutive over-expression of the Hepatitis B virus X gene (HBx) in transgenic mouse livers. Without exception, steatohepatitis was associated with reduced insulin receptor (IR) binding, IR gene expression, IR tyrosine phosphorylation, IR tyrosine kinase activation, signaling through IRS-1, and insulin responsive gene expression, and increased oxidative stress and adduct (DNA, protein, and lipid) accumulation. In human cases of NASH, insulin sensitizer drug treatments that reduce severity of steatohepatitis increase insulin responsive gene expression in liver [62,63]. Therefore, irrespective of cause, hepatic steatosis and steatohepatitis have pivotal roles in the pathogenesis of liver insulin resistance.

Insulin stimulates lipogenesis, which results in increased triglyceride storage in the liver [64,65]. While this process is generally benign and well tolerated, disturbances in homeostasis caused by ER stress, oxidative damage, mitochondrial dysfunction, inflammation, and/or altered membrane lipid composition can shift the balance toward a state of insulin resistance [64,66]. Insulin resistance promotes lipolysis [67], and lipolysis generates toxic lipids, i.e., ceramides, which further impair insulin signaling, mitochondrial function, and cell viability [66,68,69]. We propose that this sequence of events establishes a reverberating loop of progressive hepatic dysfunction that could evolve toward end-stage liver disease.

### Ceramides as Culprits

Ceramides are lipid signaling molecules that modulate positive or negative cellular responses such as, proliferation, motility, plasticity, inflammation, apoptosis, and insulin resistance [70]. Lipids stored in hepatocyes are mobilized by lipolysis and degradation of sphingomyelin. Ceramides are generated during biosynthesis and degradation of triglycerides and sphingomyelin [68,71–74]. Disease-associated lipolysis is a feature of insulin resistance, and can be initiated by critically high levels of ER stress and mt dysfunction [75–78]. In such states, ceramides cause insulin resistance by activating pro-inflammatory cytokines and inhibiting insulin-stimulated signaling through PI3 kinase-Akt [79–82]. In diet-induced obesity, the mechanisms of enhanced ceramide production in adipocytes and attendant insulin resistance have been well documented [70,73,74,83–85]. In contrast, there is little information about the role of ceramides in relation to hepatic insulin resistance. To address this issue, we conducted experiments to determine whether: 1) ceramide exposure causes hepatic insulin resistance; 2) chronic ethanol exposure or other models of steatohepatitis lead to increased pro-ceramide gene expression in liver; and 3) hepatic steatosis and steatohepatitis result in increased ceramide levels (immunoreactivity) in liver and serum. The studies demonstrated that in vitro treatment with C2 or C6 synthetic ceramide significantly impairs hepatocellular viability, mt function, and insulin-stimulated signaling through Akt and GSK-3β. Therefore, exogenous ceramide exposure is hepatotoxic and causes hepatic insulin resistance.

## 5. Factors Regulating Ceramide Biosynthesis and Accumulation in Liver

**Figure 3.**
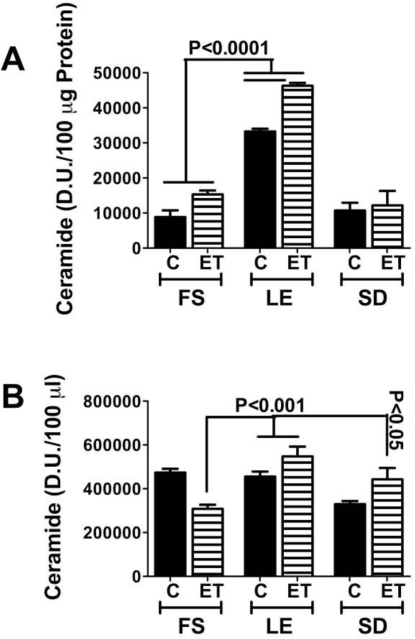
Genetic factors governing degrees of alcohol-induced hepatic steatosis and neurodegeneration correlate with liver and serum ceramide content as measured by ELISA or dot blot analysis. Immunoreactivity was detected with HRP conjugated secondary antibody and enhanced chemiluminescence reagents, and quantified by digital imaging. Significant P-values are indicated over the bars.

Ceramides are enzymatically generated through the actions of several genes whose regulations are still being investigated. To assess the potential role of ceramides in the pathogenesis of insulin resistance in the context of hepatic steatosis or steatohepatitis, we used quantitative (q) RT-PCR to measure pro-ceramide mRNA levels in several models of steatohepatitis including ASH (rat), NASH (rat, mouse, human), and DIO (rat). Irrespective of the etiology of steatohepatitis, the mean levels of several pro-ceramide mRNA transcripts were significantly increased over control. In addition, by both dot blot analysis and ELISA, we detected significantly higher levels of ceramide immunoreactivity in liver and serum of ASH, NASH, and DIO models, and in humans with NASH. Moreover, recent studies demonstrated that hepatic and serum ceramide levels correlate with severity of alcohol-induced steatohepatitis in the LE, SD, and FS rat strains (Figure 3). These studies demonstrate that pro-ceramide gene expression and hepatic and serum ceramide levels increase with steatohepatitis from various causes.

### Endogenous Brain Ceramide Production

Many studies, including our own, have demonstrated direct toxic and degenerative effects of ethanol using in vitro models [51,52,58,59]. Therefore, ethanol capable of bypassing peripheral detoxification systems can directly cause CNS injury and degeneration by increasing oxidative stress, DNA damage, lipid peroxidation, and mt dysfunction, and perturbing membrane lipid composition, leading to increased insulin/IGF resistance [33,34,36]. CNS neurons and oligodendrocytes are insulin-responsive [17,22,26] Insulin and IGF promote viability, energy metabolism, neurotransmitter synthesis, plasticity, and myelin homeostasis in these cell types. Metabolic stresses impair oligodendroglial functions, including myelin maintenance [23,27,29]. Since ceramides are generated in brain during myelin turnover and degradation, factors that impair oligodendroglial function would be expected to increase ceramide synthesis [86–90]. Locally increased ceramide production would worsen brain insulin/IGF resistance, neuro-inflammation, and oxidative stress [70,72,74,80,85,91–95]. The degree to which this scenario mediates alcohol-related neurodegeneration, i.e., white matter atrophy and leukoencephalopathy, would likely correlate with inefficiency of peripheral (gastrointestinal and hepatic) detoxification systems or binge exposures that overwhelm the network. In essence, alcohol-induced hepatotoxicity with increased generation of peripheral ceramides and other toxic lipids, would likely mediate their adverse effects on CNS structure and function via a liver-brain axis of neurodegeneration.

**Figure 4.**
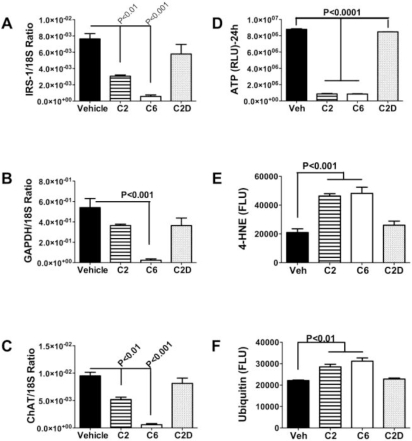
Ceramides cause insulin resistance, acetylcholine deficits, energy depletion, and oxidative stress. Cultured CNS neuronal cells were exposed to vehicle (Veh), C2 or C6 cytotoxic ceramides (50 mM), or C2D inactive ceramide for 24 hours. Gene expression was measured by qRT-PCR with results normalized to 18S rRNA. As indices of oxidative stress, (E) 4-hydroxynonenal (4-HNE) and (F) ubiquitin immunoreactivity were measured by ELISA [96].

Correspondingly, exogenous exposure to neurotoxic ceramides results in neuronal insulin resistance, neurodegeneration, energy failure, and increased oxidative stress (Figure 4).

## 6. Liver Brain Axis of Neurodegeneration

### Ceramides and the Liver-Brain Axis of Neurodegeneration

**Figure 5.**
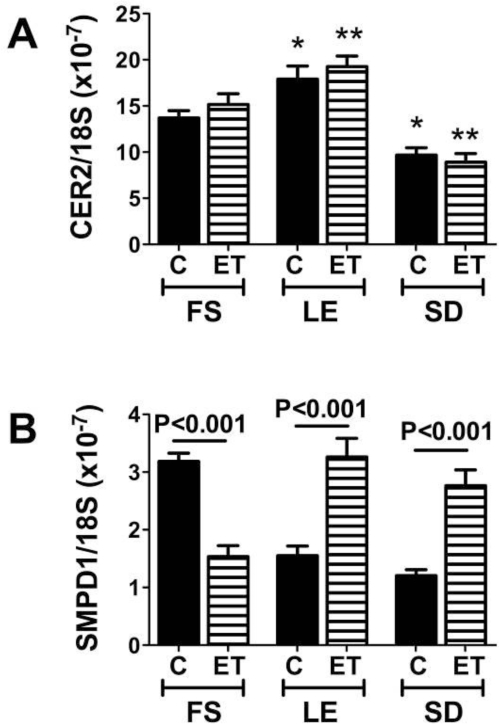
Genetic factors govern shifts in hepatic ceramide gene expression levels associated with chronic ethanol exposure. FS, LE, and SD rats were chronically fed with control or ethanol-containing liquid diets for 8 weeks, and then livers were used to measure pro-ceramide gene expression by qRT-PCR. Example results are shown for (A) Ceramide Synthase 2 (Cer2) and (B) sphingomyelinase 1 (SMPD1). Significant P-values are indicated over the bars. In addition, asterisks reflect significant differences from FS controls (*P < 0.05; **P < 0.01). Similar results have been obtained using various models of hepatic steatosis/steatohepatitis [107,110,111].

Clinically, ASH, NASH, and viral steatohepatitis can each be associated with cognitive and neuropsychiatric dysfunction [97–103]. We examined insulin signaling mechanisms, pro-ceramide gene expression, and ceramide levels in experimental models of steatohepatitis or hepatic steatosis, including chronic HFD feeding, peripheral (i.p.) nitrosamine exposure (sub-mutagenic doses), and DIO with T2DM and NASH, all of which have histopathologic and biochemical evidence of neurodegeneration, and deficits in learning and memory by Morris water maze testing [104–108]. Steatohepatitis was associated with liver and brain insulin resistance manifested by reduced insulin receptor (IR) binding, IR gene expression, IR tyrosine kinase activation, and insulin responsive gene expression, including those required for metabolism or neurotransmitter synthesis [104–110], and increased oxidative stress. Using qRT-PCR analysis, we found that steatohepatitis was associated with up-regulated expression of multiple pro-ceramide genes in liver (Figure 5). ELISA and dot blot analyses demonstrated higher mean levels of ceramide immunoreactivity in liver and blood.

**Figure 6.**
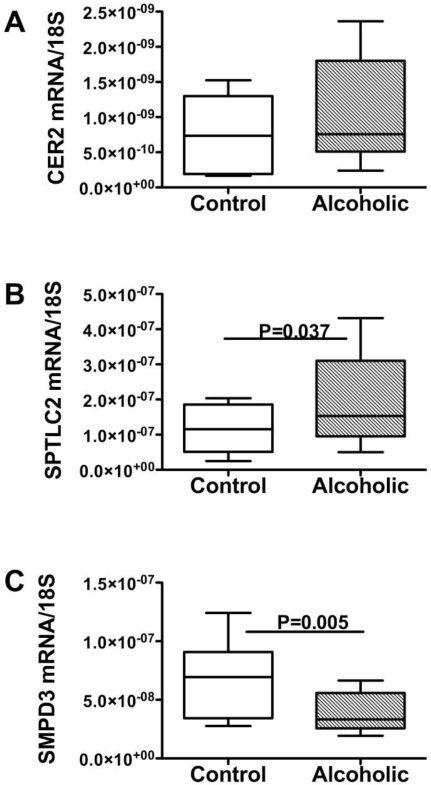
Pro-ceramide gene expression in human controls and chronic alcoholic brains as measured by qRT-PCR analysis. RNA was isolated from frontal lobes of postmortem brains and used to measure (A) ceramide synthase 2 (CER2), (B) SPTLC2, and (C) Sphingomyelinase 3 (SMPD3). Results were normalize to 18S rRNA. Significant P-values are shown over the bars. CER2 and SPTLC2 generate ceramide via a biosynthetic pathway. SPMD3 produces ceramides and other toxic lipids via a degradation pathway [35].

Ethanol-fed rats with steatohepatitis developed CNS insulin resistance with neurodegeneration and cognitive impairment [11,12,16], but in that model, pro-ceramide gene expression was increased in both liver and brain. In human alcoholics, white matter atrophy and degeneration with reduced expression of myelin-associated genes, and increased levels of oxidative stress were associated with increased expression of pro-ceramide genes (Figure 6) [35]. So, how could this be explained?

Since toxic lipids, including ceramides readily cross the blood-brain barrier and cause insulin resistance by interfering with critical phosphorylation events [112–114] and activating pro-inflammatory cytokines [70,115,116], we conducted several experiments designed to address our hypothesis about the potential role of extra-CNS (liver)-derived ceramides as mediators of neurodegeneration. *In vitro* experiments demonstrated that C2 or C6 ceramide exposures cause neuronal insulin resistance with reduced viability and neurotransmitter function, and increased mitochondrial dysfunction, oxidative stress, DNA damage, and lipid peroxidation (Figure 4) [96]. Moreover, preliminary studies showed that *in vivo* (i.p.) ceramide treatments cause brain insulin resistance, neurodegeneration, cognitive-motor deficits that mimic features of chronic alcohol feeding, and later increased pro-ceramide gene expression in brain. Therefore, ceramides generated or delivered from extra-CNS sources can cause brain insulin resistance and thereby mediate neurodegeneration. In addition, exposure to exogenous ceramides could increase CNS expression of pro-ceramide genes.

## 7. Conclusions

Chronic alcohol exposure resulting in steatohepatitis, ER stress, and persistent hepatic injury, promotes lipolysis and increased generation of toxic lipids, including ceramides. Ceramides exacerbate liver injury by causing insulin resistance, oxidative stress, and pro-inflammatory cytokine activation. Ceramides that are released into peripheral blood, and lipid soluble and therefore can readily cross the blood-brain barrier. In the CNS, ceramides initiate a complex cascade of neurodegeneration mediated by insulin resistance, inflammation, and oxidative stress. CNS insulin resistance leads to neuronal loss and impaired oligodendrocyte function. Attendant myelin degradation leads to increased CNS toxic lipid production and worsening of neurodegeneration. In addition, direct neurotoxic effects of alcohol and its metabolites, which impair mitochondrial function, reduce membrane integrity, and increase oxidative stress also contributes to neurodegeneration.

**Figure 7.**
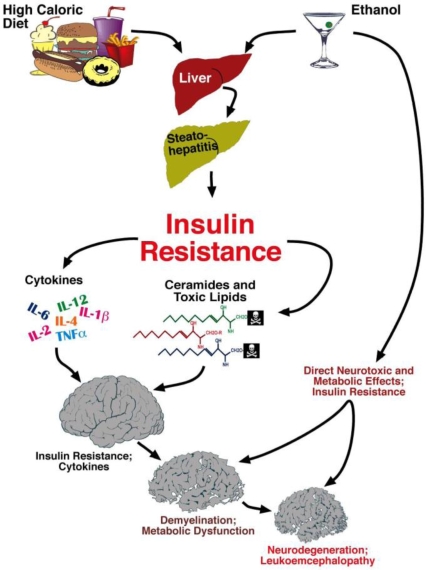
Liver-brain axis of neurodegeneration—Hypothesis. Progressive hepatic steatosis incites inflammation and pro-inflammatory cytokine activation. Attendant insulin resistance initiates a lipolysis and lipid disequilibrium cascade, leading to increased production and accumulation of ceramides. Ceramides are cytotoxic and promote insulin resistance, and their lipid solubility enables ready transfer across the blood-brain barrier to cause CNS insulin resistance and neurodegeneration with loss of neurons and oligodendrocytes. Ethanol’s lipid solubility enables it to exert direct neurotoxic effects, and cause brain insulin resistance.

Brain insulin resistance leads to neuronal loss and impaired neurotransmitter function required for plasticity, learning, and memory. In addition, brain insulin resistance impairs oligodendrocyte survival and function, resulting in reduced myelin integrity, and increased generation of ceramides, which further increase brain insulin resistance, neuro-inflammation, oxidative stress, and neurodegeneration. These concepts open an exciting new chapter on disease mechanisms and strategies for developing non-invasive tools to monitor proneness and progression of alcoholic neurodegeneration. Our over-arching hypothesis about how alcohol mis-use causes neurodegeneration, both directly and via the liver-brain axis is diagramed in Figure 7. It is noteworthy that dietary indiscretions leading to hepatic steatosis, i.e., chronic high caloric intake, significantly compound these problems and contribute to the liver-brain axis of neurodegeneration.
